# On some nonlinear retarded Volterra–Fredholm type integral inequalities on time scales and their applications

**DOI:** 10.1186/s13660-018-1808-6

**Published:** 2018-08-15

**Authors:** Haidong Liu

**Affiliations:** School of Mathematical Sciences, Qufu Normal University, Qufu, P.R. China

**Keywords:** Time scale, Retarded integral inequality, Volterra–Fredholm type

## Abstract

In this paper, we establish some new nonlinear retarded Volterra–Fredholm type integral inequalities on time scales. Our results not only generalize and extend some known integral inequalities, but also provide a handy and effective tool for the study of qualitative properties of solutions of some Volterra–Fredholm type dynamic equations.

## Introduction

In recent years, there exist a large number of published papers on the theory of time scales which was introduced by Stefan Hilger [1] in his Ph.D. thesis in 1988 in order to unify and extend the difference and differential calculus in a consistent way, for instance, [2–16] and the references therein. In particular, many scholars attached great importance to the study of dynamic inequalities on time scales (see, e.g., [17–31] and the references therein), which extended some discrete and continuous inequalities (see [32–36] and the references therein).

In 2013, the authors in [22] established and applied the following useful linear Volterra–Fredholm type integral inequality on time scales:

$$ u(t) \leq u_{0}+ \int_{t_{0}}^{t}f(\tau) \biggl[u(\tau)+ \int _{t_{0}}^{\tau}g(s)u(s)\Delta s \biggr] \Delta\tau+ \int_{t_{0}}^{\alpha}h(\tau)u(\tau)\Delta\tau,\quad t\in I, $$

where $I=[t_{0},\alpha]\cap\mathbb{T}$, $t_{0}\in\mathbb{T}$, $\alpha\in \mathbb{T}$, $\alpha>t_{0}$, $u_{0}$ is a nonnegative constant, *u*, *f*, *g*, and *h* are nonnegative rd-continuous functions defined on *I*.

In 2014, the authors in [27] investigated the nonlinear Volterra–Fredholm type integral inequality on time scales

$$\begin{aligned} u(t) \leq &k + \int^{t}_{t_{0}}f_{1}(s)w\bigl(u(s)\bigr) \Delta s+ \int ^{t}_{t_{0}}f_{2}(s) \int^{s}_{t_{0}}f_{3}(\tau)w\bigl(u(\tau) \bigr)\Delta\tau\Delta s \\ &{} + \int^{\alpha}_{t_{0}}f_{1}(s)w\bigl(u(s)\bigr) \Delta s+ \int^{\alpha }_{t_{0}}f_{2}(s) \int^{s}_{t_{0}}f_{3}(\tau)w\bigl(u(\tau) \bigr)\Delta\tau\Delta s , \quad t\in I, \end{aligned}$$

where $I=[t_{0},\alpha]\cap\mathbb{T}$, $t_{0}\in\mathbb{T}$, $\alpha\in \mathbb{T}$, $\alpha>t_{0}$, *u*, $f_{1}$, $f_{2}$, $f_{3}$ are rd-continuous functions defined on *I*, $f_{1}$, $f_{2}$, $f_{3}$ are nonnegative, $w\in C(\mathbb{R}_{+}, \mathbb{R}_{+})$ is a nondecreasing function with $w(u)>0$ for $u>0$, and *k* is a nonnegative constant.

Very recently, the author in [29] discovered the retarded Volterra–Fredholm type integral inequality on time scales

$$\begin{aligned} u(t) \leq& a(t) + b(t) \int_{\alpha(t_{0})}^{\alpha(t)} \biggl[f_{1}(s)u(s)+f_{2}(s) \int_{\alpha(t_{0})}^{s}g(\tau)u(\tau)\Delta\tau \biggr] \Delta s \\ &{} + \lambda b(T) \int_{\alpha(t_{0})}^{\alpha(T)} \biggl[f_{1}(s)u(s)+f_{2}(s) \int _{\alpha(t_{0})}^{s}g(\tau)u(\tau)\Delta\tau \biggr] \Delta s,\quad t\in I, \end{aligned}$$

where $I=[t_{0},T]\cap\mathbb{T}$, $t_{0}\in\mathbb{T}$, $T\in\mathbb{T}$, $T>t_{0}$, $\alpha:I\rightarrow I$ is continuous and strictly increasing satisfying $\alpha(t)\leq t$, $\alpha^{\Delta}$ is rd-continuous, *u*, *a*, *b*, *f*, and $g: I\rightarrow\mathbb{R}_{+}$ are rd-continuous functions and *a* is nondecreasing.

Inspired by the ideas employed in [22, 27, 29], here we obtain some new nonlinear Volterra–Fredholm type integral inequalities on time scales. Our results not only generalize and extend the results of [22, 27] and some known integral inequalities but also provide a handy and effective tool for the study of qualitative properties of solutions of some complicated Volterra–Fredholm type dynamic equations.

## Preliminaries

For an excellent introduction to the calculus on time scales, we refer the reader to [5] and [6].

In what follows, we always assume that $\mathbb{R}$ denotes the set of real numbers, $\mathbb{R}_{+}=[0,\infty)$, $\mathbb{Z}$ denotes the set of integers, and $\mathbb{T}$ is an arbitrary time scale (nonempty closed subset of $\mathbb{R}$), $\mathcal{R}$ denotes the set of all regressive and rd-continuous functions, $\mathcal{R}^{+}=\{p\in\mathcal{R}:1+\mu (t)p(t)>0, \mbox{for all } t\in\mathbb{T}\}$, $I=[t_{0},T]\cap\mathbb{T}^{\kappa}$, where $t_{0}\in\mathbb{T}^{\kappa}$, $T\in\mathbb{T}^{\kappa}$, $T>t_{0}$. The set $\mathbb{T}^{\kappa}$ is defined as follows: If $\mathbb{T}$ has a maximum *m* and *m* is left-scattered, then $\mathbb{T}^{\kappa}=\mathbb{T}-\{m\}$. Otherwise $\mathbb{T}^{\kappa}=\mathbb{T}$. The graininess function $\mu: \mathbb {T}\rightarrow[0,\infty)$ is defined by $\mu(t):=\sigma(t)-t$, the forward jump operator $\sigma: \mathbb{T}\rightarrow\mathbb{T}$ by $\sigma(t):=\inf\{s\in\mathbb{T}: s>t\}$, and the “circle plus” addition ⊕ defined by $(p\oplus q)(t):=p(t)+q(t)+\mu(t)p(t)q(t)$ for all $t\in\mathbb {T}^{\kappa}$.

We give the following lemmas in order to use them in our proofs. One can find details in [5].

### Lemma 2.1

([5, Theorem 1.16])

*Assume that* $f:\mathbb{T}\rightarrow\mathbb{R}$ *is a function and let* $t\in\mathbb{T}$. *If* *f* *is differentiable at* *t*, *then*

$$f\bigl(\sigma(t)\bigr)=f(t)+\mu(t)f^{\Delta}(t). $$

### Lemma 2.2

([5, Theorem 1.98])

*Assume that* $\nu:\mathbb {T}\rightarrow\mathbb{R}$ *is a strictly increasing function and* $\widetilde{\mathbb{T}}:=\nu(\mathbb{T})$ *is a time scale*. *If* $f: \mathbb{T}\rightarrow\mathbb{R}$ *is an rd*-*continuous function and* *ν* *is differentiable with rd*-*continuous derivative*, *then for* $a, b\in \mathbb{T}$,

$$\int_{a}^{b}f(t)\nu^{\Delta}(t)\Delta t= \int_{\nu(a)}^{\nu(b)}\bigl(f\circ\nu ^{-1}\bigr) (s)\widetilde{\Delta} s. $$

### Lemma 2.3

([29])

*Let* $\alpha:I\rightarrow I$ *be a continuous and strictly increasing function such that* $\alpha(t)\leq t$, *and* $\alpha^{\Delta}$ *is rd*-*continuous*. *Assume that* $f: I\rightarrow\mathbb{R}$ *is an rd*-*continuous function*, *then*

2.1 $$ g(t)= \int_{\alpha(t_{0})}^{\alpha(t)}f(s)\Delta s, \quad t\in I, $$

*implies*

2.2 $$ g^{\Delta}(t)=f\bigl(\alpha(t)\bigr)\alpha^{\Delta}(t),\quad t\in I. $$

### Lemma 2.4

([5, Theorem 1.117])

*Suppose that for each* $\varepsilon>0$ *there exists a neighborhood* *U* *of* *t*, *independent of* $\tau\in[t_{0},\sigma(t)]$, *such that*

2.3 $$ \bigl\vert w\bigl(\sigma(t),\tau\bigr)-w(s,\tau) -w^{\Delta}_{t}(t, \tau) \bigl(\sigma(t)-s\bigr) \bigr\vert \leq\varepsilon \bigl\vert \sigma(t)-s \bigr\vert , \quad s\in U, $$

*where* $w: \mathbb{T}\times\mathbb{T}^{\kappa}\rightarrow\mathbb{R}_{+}$ *is continuous at* $(t,t)$, $t\in\mathbb{T}^{\kappa}$ *with* $t>t_{0}$, *and* $w^{\Delta}_{t}(t,\cdot)$ *are rd*-*continuous on* $[t_{0},\sigma(t)]$. *Then*

$$g(t):= \int_{t_{0}}^{t}w(t,\tau)\Delta\tau $$

*implies*

2.4 $$ g^{\Delta}(t)= \int_{t_{0}}^{t}w^{\Delta}_{t}(t,\tau) \Delta\tau+w\bigl(\sigma(t),t\bigr),\quad t\in\mathbb{T}^{\kappa}. $$

### Lemma 2.5

([5, Theorem 6.1])

*Suppose that* *y* *and* *f* *are rd*-*continuous functions and* $p\in\mathcal {R}^{+}$. *Then*

$$y^{\Delta}(t)\leq p(t)y(t)+f(t),\quad t \in\mathbb{T} $$

*implies*

$$y(t)\leq y(t_{0})e_{p}(t,t_{0})+ \int_{t_{0}}^{t}e_{p}\bigl(t,\sigma(\tau) \bigr)f(\tau)\Delta \tau,\quad t \in\mathbb{T}. $$

### Lemma 2.6

*Let* $m\geq n\geq0$, $m\neq0$, *and* $a\geq0$, *then*

2.5 $$ a^{n}\leq{\frac{n}{m}}k^{n-m}a^{m}+ \frac{m-n}{m}k^{n} $$

*for any* $k>0$.

### Proof

Set $F(x)={\frac{n}{m}}x^{n-m}a^{m}+\frac{m-n}{m}x^{n}$, $x>0$. It is seen that $F(x)$ obtains its minimum at $x_{0}=a$. Hence we get (2.5) holds for any $k>0$. □

Throughout this paper, we assume that:

($\mathrm{H}_{1}$)
$\alpha:I\rightarrow I$ is continuous and strictly increasing satisfying $\alpha(t)\leq t$ and $\alpha^{\Delta}$ is rd-continuous.
($\mathrm{H}_{2}$)
$\beta, \gamma:I\rightarrow I$ are continuous satisfying $\beta (t)\leq t$ and $\gamma(t)\leq t$.
($\mathrm{H}_{3}$)
$u, a, b: I\rightarrow\mathbb{R}_{+}$ are rd-continuous functions, *a* is nondecreasing, and $b^{\Delta}(t)\geq0$.
($\mathrm{H}_{4}$)
$f_{i}\ (i=1,2,3,4,5,6), g_{i}\ (i=1,2,3): I\rightarrow\mathbb{R}_{+}$ are rd-continuous functions.
($\mathrm{H}_{5}$)
$v, w: \mathbb{T}\times\mathbb{T}^{\kappa}\rightarrow\mathbb {R}_{+}$ is continuous at $(t,t)$, $t\in\mathbb{T}^{\kappa}$ with $t>t_{0}$.
($\mathrm{H}_{6}$)
$m, n: \mathbb{T}\times\mathbb{T}^{\kappa}\rightarrow\mathbb {R}_{+}$ is continuous at $(t,t)$, $t\in\mathbb{T}^{\kappa}$ with $t>t_{0}$.

## Main results

### Theorem 3.1

*Assume that* ($\mathrm{H}_{1}$)*–*($\mathrm{H}_{4}$) *hold*, $0\leq p\leq1$, $0\leq q\leq1$, $0\leq r\leq1$ *are constants*, *and* $\mu(t)A(t)<1$. *Suppose that* *u* *satisfies*

3.1 $$\begin{aligned} u(t) \leq& a(t) + b(t) \int_{\alpha(t_{0})}^{\alpha (t)}f_{1}(s) \biggl[u(s)+f_{2}(s) \int_{\alpha(t_{0})}^{s}g_{1}(\tau)u(\tau)\Delta \tau \biggr]^{p} \Delta s \\ &{} + \int_{\beta(t_{0})}^{\beta(T)}f_{3}(s) \biggl[u(s)+f_{4}(s) \int_{\beta (t_{0})}^{s}g_{2}(\tau)u(\tau)\Delta\tau \biggr]^{q} \Delta s \\ &{} + \int_{\gamma(t_{0})}^{\gamma(T)}f_{5}(s) \biggl[u(s)+f_{6}(s) \int_{\gamma (t_{0})}^{s}g_{3}(\tau)u(\tau)\Delta\tau \biggr]^{r} \Delta s, \quad t\in I. \end{aligned}$$

*If there exist positive constants* $k_{i}$ ($i=1,2,3$) *such that*

3.2 $$\begin{aligned} \lambda :=& \int_{\beta(t_{0})}^{\beta(T)} \biggl[qk_{2}^{q-1}f_{3}(s) \biggl(e_{B \oplus C}(s,t_{0})+f_{4}(s) \int_{\beta (t_{0})}^{s}g_{2}(\tau)e_{B \oplus C}( \tau,t_{0})\Delta\tau \biggr) \biggr] \Delta s \\ &{}+ \int_{\gamma(t_{0})}^{\gamma(T)} \biggl[rk_{3}^{r-1}f_{5}(s) \biggl(e_{B \oplus C}(s,t_{0})+f_{6}(s) \int_{\gamma(t_{0})}^{s}g_{3}(\tau)e_{B \oplus C}( \tau ,t_{0})\Delta\tau \biggr) \biggr] \Delta s< 1, \end{aligned}$$

*then*

3.3 $$ u(t) \leq \frac{K+V(T)}{1-\lambda}e_{B \oplus C}(t,t_{0}),\quad t\in I, $$

*where*

3.4 $$\begin{aligned}& K = \int_{\beta(t_{0})}^{\beta(T)}(1-q)k_{2}^{q}f_{3}(s) \Delta s+ \int_{\gamma(t_{0})}^{\gamma(T)}(1-r)k_{3}^{r}f_{5}(s) \Delta s, \end{aligned}$$

3.5 $$\begin{aligned}& V(t) = a(t)+b(t) \int_{\alpha(t_{0})}^{\alpha(t)}(1-p)k_{1}^{p}f_{1}(s) \Delta s, \end{aligned}$$

3.6 $$\begin{aligned}& A(t) = b^{\Delta}(t) \int_{\alpha(t_{0})}^{\alpha(\sigma(t))} \biggl[pk_{1}^{p-1}f_{1}(s) \biggl(1+f_{2}(s) \int_{\alpha(t_{0})}^{s}g_{1}(\tau)\Delta\tau \biggr) \biggr] \Delta s, \end{aligned}$$

3.7 $$\begin{aligned}& B(t) = \frac{A(t)}{1-\mu(t)A(t)}, \end{aligned}$$

3.8 $$\begin{aligned}& C(t) = b(t) \biggl[pk_{1}^{p-1}f_{1}\bigl( \alpha(t)\bigr) \biggl(1+f_{2}\bigl(\alpha(t)\bigr) \int _{\alpha(t_{0})}^{\alpha(t)}g_{1}(\tau)\Delta\tau \biggr) \biggr]\alpha^{\Delta}(t). \end{aligned}$$

### Proof

Denote

3.9 $$\begin{aligned} z(t) =&a(t)+b(t) \int_{\alpha(t_{0})}^{\alpha (t)}f_{1}(s) \biggl[u(s)+f_{2}(s) \int_{\alpha(t_{0})}^{s}g_{1}(\tau)u(\tau)\Delta \tau \biggr]^{p} \Delta s \\ &{}+ \int_{\beta(t_{0})}^{\beta(T)}f_{3}(s) \biggl[u(s)+f_{4}(s) \int_{\beta (t_{0})}^{s}g_{2}(\tau)u(\tau)\Delta\tau \biggr]^{q} \Delta s \\ &{}+ \int_{\gamma(t_{0})}^{\gamma(T)}f_{5}(s) \biggl[u(s)+f_{6}(s) \int_{\gamma (t_{0})}^{s}g_{3}(\tau)u(\tau)\Delta\tau \biggr]^{r} \Delta s, \quad t\in I. \end{aligned}$$

Then *z* is nondecreasing on *I*. From (3.1) and (3.9) we have

3.10 $$ u(t)\leq z(t),\quad t\in I. $$

Now using Lemma 2.6 for $a=u(s)+f_{i}(s)\int_{\alpha(t_{0})}^{s}g_{j}(\tau )u(\tau)\Delta\tau$, $i=2,4,6$, and $j=i/2$ with $m=1$ and $n=p,q,r$ for any $k_{1}, k_{2}, k_{3}>0$, respectively, we have

3.11 $$\begin{aligned} z(t) \leq&a(t)+b(t) \int_{\alpha(t_{0})}^{\alpha(t)} \biggl\{ pk_{1}^{p-1}f_{1}(s) \biggl[u(s)+f_{2}(s) \int_{\alpha(t_{0})}^{s}g_{1}(\tau)u(\tau )\Delta \tau \biggr] \\ &{}+(1-p)k_{1}^{p}f_{1}(s) \biggr\} \Delta s \\ &{}+ \int_{\beta(t_{0})}^{\beta(T)} \biggl\{ qk_{2}^{q-1}f_{3}(s) \biggl[u(s)+f_{4}(s) \int_{\beta(t_{0})}^{s}g_{2}(\tau)u(\tau)\Delta\tau \biggr] \\ &{}+(1-q)k_{2}^{q}f_{3}(s) \biggr\} \Delta s \\ &{}+ \int_{\gamma(t_{0})}^{\gamma(T)} \biggl\{ rk_{3}^{r-1}f_{5}(s) \biggl[u(s)+f_{6}(s) \int_{\gamma(t_{0})}^{s}g_{3}(\tau)u(\tau)\Delta\tau \biggr] \\ &{}+(1-r)k_{3}^{r}f_{5}(s) \biggr\} \Delta s. \end{aligned}$$

Now using (3.4) and (3.5) and (3.10) we get

3.12 $$\begin{aligned} z(t) \leq& K+V(t)+b(t) \int_{\alpha(t_{0})}^{\alpha(t)} \biggl\{ pk_{1}^{p-1}f_{1}(s) \biggl[z(s) \\ &{}+f_{2}(s) \int_{\alpha(t_{0})}^{s}g_{1}(\tau)z(\tau)\Delta\tau \biggr] \biggr\} \Delta s \\ &{}+ \int_{\beta(t_{0})}^{\beta(T)} \biggl\{ qk_{2}^{q-1}f_{3}(s) \biggl[z(s)+f_{4}(s) \int_{\beta(t_{0})}^{s}g_{2}(\tau)z(\tau)\Delta\tau \biggr] \biggr\} \Delta s \\ &{}+ \int_{\gamma(t_{0})}^{\gamma(T)} \biggl\{ rk_{3}^{r-1}f_{5}(s) \biggl[z(s)+f_{6}(s) \int_{\gamma(t_{0})}^{s}g_{3}(\tau)z(\tau)\Delta\tau \biggr] \biggr\} \Delta s,\quad t\in I. \end{aligned}$$

Since $V(t)$ is nondecreasing on *I*, then for $t\in I$, from the above inequality we have

3.13 $$\begin{aligned} z(t) \leq& K+V(T)+b(t) \int_{\alpha(t_{0})}^{\alpha(t)} \biggl[pk_{1}^{p-1}f_{1}(s) \biggl(z(s)+f_{2}(s) \int_{\alpha(t_{0})}^{s}g_{1}(\tau)z(\tau )\Delta \tau \biggr) \biggr] \Delta s \\ &{}+ \int_{\beta(t_{0})}^{\beta(T)} \biggl[qk_{2}^{q-1}f_{3}(s) \biggl(z(s)+f_{4}(s) \int _{\beta(t_{0})}^{s}g_{2}(\tau)z(\tau)\Delta\tau \biggr) \biggr] \Delta s \\ &{}+ \int_{\gamma(t_{0})}^{\gamma(T)} \biggl[rk_{3}^{r-1}f_{5}(s) \biggl(z(s)+f_{6}(s) \int_{\gamma(t_{0})}^{s}g_{3}(\tau)z(\tau)\Delta\tau \biggr) \biggr] \Delta s,\quad t\in I. \end{aligned}$$

Let

3.14 $$\begin{aligned} M =&K+V(T)+ \int_{\beta(t_{0})}^{\beta(T)} \biggl[qk_{2}^{q-1}f_{3}(s) \biggl(z(s)+f_{4}(s) \int_{\beta(t_{0})}^{s}g_{2}(\tau)z(\tau)\Delta\tau \biggr) \biggr] \Delta s \\ &{}+ \int_{\gamma(t_{0})}^{\gamma(T)} \biggl[rk_{3}^{r-1}f_{5}(s) \biggl(z(s)+f_{6}(s) \int_{\gamma(t_{0})}^{s}g_{3}(\tau)z(\tau)\Delta\tau \biggr) \biggr] \Delta s. \end{aligned}$$

Then (3.13) can be restated as

3.15 $$ z(t)\leq M+b(t) \int_{\alpha(t_{0})}^{\alpha(t)} \biggl[pk_{1}^{p-1}f_{1}(s) \biggl(z(s)+f_{2}(s) \int_{\alpha(t_{0})}^{s}g_{1}(\tau)z(\tau)\Delta\tau \biggr) \biggr] \Delta s,\quad t\in I. $$

Set

3.16 $$ w(t)=M+b(t) \int_{\alpha(t_{0})}^{\alpha(t)} \biggl[pk_{1}^{p-1}f_{1}(s) \biggl(z(s)+f_{2}(s) \int_{\alpha(t_{0})}^{s}g_{1}(\tau)z(\tau)\Delta\tau \biggr) \biggr] \Delta s, \quad t\in I. $$

Then $w(t)$ is nondecreasing, and from (3.15) and (3.16) we obtain

3.17 $$ z(t)\leq w(t),\quad t\in I. $$

Using Lemma 2.3, taking delta derivative of (3.16), and from (3.17), we have

3.18 $$\begin{aligned} w^{\Delta}(t) =&b^{\Delta}(t) \int_{\alpha(t_{0})}^{\alpha (\sigma(t))} \biggl[pk_{1}^{p-1}f_{1}(s) \biggl(z(s)+f_{2}(s) \int_{\alpha (t_{0})}^{s}g_{1}(\tau)z(\tau)\Delta\tau \biggr) \biggr] \Delta s \\ &{}+b(t) \biggl[pk_{1}^{p-1}f_{1}\bigl(\alpha(t) \bigr) \biggl(z\bigl(\alpha(t)\bigr)+f_{2}\bigl(\alpha(t)\bigr) \int _{\alpha(t_{0})}^{\alpha(t)}g_{1}(\tau)z(\tau)\Delta \tau \biggr) \biggr]\alpha ^{\Delta}(t) \\ \leq&b^{\Delta}(t) \int_{\alpha(t_{0})}^{\alpha(\sigma(t))} \biggl[pk_{1}^{p-1}f_{1}(s) \biggl(w(s)+f_{2}(s) \int_{\alpha(t_{0})}^{s}g_{1}(\tau)w(\tau )\Delta \tau \biggr) \biggr] \Delta s \\ &{}+b(t) \biggl[pk_{1}^{p-1}f_{1}\bigl(\alpha(t) \bigr) \biggl(w\bigl(\alpha(t)\bigr)+f_{2}\bigl(\alpha(t)\bigr) \int _{\alpha(t_{0})}^{\alpha(t)}g_{1}(\tau)w(\tau)\Delta \tau \biggr) \biggr]\alpha ^{\Delta}(t) \\ \leq&w\bigl(\sigma(t)\bigr)b^{\Delta}(t) \int_{\alpha(t_{0})}^{\alpha(\sigma(t))} \biggl[pk_{1}^{p-1}f_{1}(s) \biggl(1+f_{2}(s) \int_{\alpha(t_{0})}^{s}g_{1}(\tau)\Delta\tau \biggr) \biggr] \Delta s \\ &{}+w(t)b(t) \biggl[pk_{1}^{p-1}f_{1}\bigl( \alpha(t)\bigr) \biggl(1+f_{2}\bigl(\alpha(t)\bigr) \int _{\alpha(t_{0})}^{\alpha(t)}g_{1}(\tau)\Delta\tau \biggr) \biggr]\alpha^{\Delta}(t) \\ =&A(t)w\bigl(\sigma(t)\bigr)+C(t)w(t), \quad t\in I, \end{aligned}$$

where $A(t)$ and $C(t)$ are defined as in (3.6) and (3.8). From (3.7), we get

3.19 $$ A(t)=\frac{B(t)}{1+\mu(t)B(t)}, $$

and from (3.18), (3.19), and Lemma 2.1, we have

3.20 $$\begin{aligned} w^{\Delta}(t) \leq&\frac{B(t)}{1+\mu(t)B(t)}w\bigl(\sigma(t)\bigr)+C(t)w(t) \\ =&\frac{B(t)}{1+\mu(t)B(t)}\bigl[w(t)+\mu(t)w^{\Delta}(t)\bigr]+C(t)w(t), \end{aligned}$$

which yields

3.21 $$ \frac{1}{1+\mu(t)B(t)}w^{\Delta}(t)\leq \biggl[\frac{B(t)}{1+\mu (t)B(t)}+C(t) \biggr]w(t), $$

i.e.,

3.22 $$\begin{aligned} w^{\Delta}(t) \leq& \bigl[B(t)+\bigl(1+\mu(t)B(t)\bigr)C(t) \bigr]w(t) \\ =&(B\oplus C) (t)w(t), \quad t\in I. \end{aligned}$$

Note that *w* is rd-continuous and $B\oplus C\in\mathcal{R}^{+}$, from Lemma 2.5, (3.16), and (3.22), we obtain

3.23 $$ w(t)\leq w(t_{0})e_{B \oplus C}(t,t_{0})=Me_{B \oplus C}(t,t_{0}), \quad t\in I. $$

From (3.17) and (3.23), we have

3.24 $$ z(t)\leq Me_{B \oplus C}(t,t_{0}),\quad t\in I. $$

Using (3.24) on the right–hand side of (3.14) and according to (3.2), we obtain

3.25 $$ M\leq\frac{K+V(T)}{1-\lambda}. $$

From (3.24) and (3.25), we obtain

3.26 $$ z(t)\leq\frac{K+V(T)}{1-\lambda}e_{B \oplus C}(t,t_{0}),\quad t\in I. $$

Noting (3.10), we get the desired inequality (3.3). This completes the proof. □

If we take $p=q=r=1$, we can get the following corollary.

### Corollary 3.1

*Assume that* ($\mathrm{H}_{1}$)*–*($\mathrm{H}_{4}$) *hold*. *Suppose that* *u* *satisfies*

$$\begin{aligned} u(t) \leq& a(t)+b(t) \int_{\alpha(t_{0})}^{\alpha (t)}f_{1}(s) \biggl[u(s)+f_{2}(s) \int_{\alpha(t_{0})}^{s}g_{1}(\tau)u(\tau)\Delta \tau \biggr] \Delta s \\ &{}+ \int_{\beta(t_{0})}^{\beta(T)}f_{3}(s) \biggl[u(s)+f_{4}(s) \int_{\beta (t_{0})}^{s}g_{2}(\tau)u(\tau)\Delta\tau \biggr] \Delta s \\ &{}+ \int_{\gamma(t_{0})}^{\gamma(T)}f_{5}(s) \biggl[u(s)+f_{6}(s) \int_{\gamma (t_{0})}^{s}g_{3}(\tau)u(\tau)\Delta\tau \biggr] \Delta s,\quad t\in I. \end{aligned}$$

*If*

$$\begin{aligned} \lambda :=& \int_{\beta(t_{0})}^{\beta(T)} \biggl[f_{3}(s) \biggl(e_{B \oplus C}(s,t_{0})+f_{4}(s) \int_{\beta (t_{0})}^{s}g_{2}(\tau)e_{B \oplus C}( \tau,t_{0})\Delta\tau \biggr) \biggr] \Delta s \\ &{}+ \int_{\gamma(t_{0})}^{\gamma(T)} \biggl[f_{5}(s) \biggl(e_{B \oplus C}(s,t_{0})+f_{6}(s) \int_{\gamma(t_{0})}^{s}g_{3}(\tau)e_{B \oplus C}( \tau ,t_{0})\Delta\tau \biggr) \biggr] \Delta s< 1, \end{aligned}$$

*then*

$$u(t) \leq \frac{a(T)}{1-\lambda}e_{B \oplus C}(t,t_{0}),\quad t\in I, $$

*where*

$$\begin{aligned}& A(t) = b^{\Delta}(t) \int_{\alpha(t_{0})}^{\alpha(\sigma(t))} \biggl[f_{1}(s) \biggl(1+f_{2}(s) \int_{\alpha(t_{0})}^{s}g_{1}(\tau)\Delta\tau \biggr) \biggr] \Delta s, \qquad B(t)=\frac{A(t)}{1-\mu(t)A(t)}, \\& C(t) = b(t) \biggl[f_{1}\bigl(\alpha(t)\bigr) \biggl(1+f_{2} \bigl(\alpha(t)\bigr) \int_{\alpha (t_{0})}^{\alpha(t)}g_{1}(\tau)\Delta\tau \biggr) \biggr]\alpha^{\Delta}(t). \end{aligned}$$

### Theorem 3.2

*Assume that* ($\mathrm{H}_{1}$)*–*($\mathrm{H}_{4}$) *hold*, $0\leq q_{i}\leq l$, $0\leq r_{i}\leq l$, $l\neq0$, *and* $0\leq\theta_{i}\leq1$ ($i=1,2,3$) *are constants*, *and* $\mu(t)A(t)<1$. *Suppose that* *u* *satisfies*

3.27 $$\begin{aligned} u^{l}(t) \leq& a(t)+b(t) \int_{\alpha(t_{0})}^{\alpha (t)}f_{1}(s) \biggl[u^{q_{1}}(s)+f_{2}(s) \int_{\alpha(t_{0})}^{s}g_{1}(\tau )u^{r_{1}}(\tau)\Delta\tau \biggr]^{\theta_{1}} \Delta s \\ &{}+ \int_{\beta(t_{0})}^{\beta(T)}f_{3}(s) \biggl[u^{q_{2}}(s)+f_{4}(s) \int_{\beta (t_{0})}^{s}g_{2}(\tau)u^{r_{2}}( \tau)\Delta\tau \biggr]^{\theta_{2}} \Delta s \\ &{}+ \int_{\gamma(t_{0})}^{\gamma(T)}f_{5}(s) \biggl[u^{q_{3}}(s)+f_{6}(s) \int _{\gamma(t_{0})}^{s}g_{3}(\tau)u^{r_{3}}( \tau)\Delta\tau \biggr]^{\theta_{3}} \Delta s,\quad t\in I. \end{aligned}$$

*If there exist positive constants* $k_{i}$ ($i=1,2,\ldots,9$) *such that*

3.28 $$\begin{aligned} \lambda :=& \int_{\beta(t_{0})}^{\beta(T)} \biggl[{\theta_{2}}k_{4}^{{\theta_{2}}-1} \widetilde{f}_{3}(s) \biggl(e_{B \oplus C}(s,t_{0})+f_{4}(s) \int_{\beta(t_{0})}^{s}\widetilde{g}_{2}( \tau)e_{B \oplus C}(\tau,t_{0})\Delta\tau \biggr) \biggr] \Delta s \\ &{}+ \int_{\gamma(t_{0})}^{\gamma(T)} \biggl[{\theta_{3}}k_{7}^{{\theta _{3}}-1} \widetilde{f}_{5}(s) \biggl(e_{B \oplus C}(s,t_{0})+f_{6}(s) \int_{\gamma (t_{0})}^{s}\widetilde{g}_{3}( \tau)e_{B \oplus C}(\tau,t_{0})\Delta\tau \biggr) \biggr] \Delta s< 1, \end{aligned}$$

*then*

3.29 $$ u(t) \leq \biggl(\frac{\widetilde{K}+\widetilde{V}(T)}{1-\lambda}e_{B \oplus C}(t,t_{0}) \biggr)^{\frac{1}{l}}, \quad t\in I, $$

*where*

3.30 $$\begin{aligned}& \begin{aligned}[b] \widetilde{K} &= \int_{\beta(t_{0})}^{\beta(T)} \biggl\{ {\theta _{2}}k_{4}^{{\theta_{2}}-1}f_{3}(s) \biggl[\frac{l-q_{2}}{l}k_{5}^{q_{2}} +f_{4}(s) \int_{\beta(t_{0})}^{s}g_{2}(\tau) \biggl( \frac{l-r_{2}}{l}k_{6}^{r_{2}} \biggr) \Delta\tau \biggr] \\ &\quad {} +(1-{\theta_{2}})k_{4}^{\theta_{2}}f_{3}(s) \biggr\} \Delta s \\ &\quad {} + \int_{\gamma(t_{0})}^{\gamma(T)} \biggl\{ {\theta_{3}}k_{7}^{{\theta _{3}}-1}f_{5}(s) \biggl[\frac{l-q_{3}}{l}k_{8}^{q_{3}} +f_{6}(s) \int_{\gamma(t_{0})}^{s}g_{3}(\tau) \biggl( \frac{l-r_{3}}{l}k_{9}^{r_{3}} \biggr)\Delta\tau \biggr] \\ &\quad {} +(1-{\theta_{3}})k_{7}^{\theta_{3}}f_{5}(s) \biggr\} \Delta s, \end{aligned} \end{aligned}$$

3.31 $$\begin{aligned}& \begin{aligned}[b] \widetilde{V}(t) &= a(t)+b(t) \int_{\alpha(t_{0})}^{\alpha(t)} \biggl\{ {\theta _{1}}k_{1}^{{\theta_{1}}-1}f_{1}(s) \biggl[\frac{l-q_{1}}{l}k_{2}^{q_{1}} +f_{2}(s) \int_{\alpha(t_{0})}^{s}g_{1}(\tau) \biggl( \frac{l-r_{1}}{l}k_{3}^{r_{1}} \biggr) \Delta\tau \biggr] \\ &\quad {} +(1-{\theta_{1}})k_{1}^{\theta_{1}}f_{1}(s) \biggr\} \Delta s, \end{aligned} \end{aligned}$$

3.32 $$\begin{aligned}& A(t) = b^{\Delta}(t) \int_{\alpha(t_{0})}^{\alpha(\sigma(t))} \biggl[{\theta _{1}}k_{1}^{{\theta_{1}}-1} \widetilde{f}_{1}(s) \biggl(1+f_{2}(s) \int_{\alpha (t_{0})}^{s}\widetilde{g}_{1}(\tau) \Delta\tau \biggr) \biggr] \Delta s, \end{aligned}$$

3.33 $$\begin{aligned}& B(t) = \frac{A(t)}{1-\mu(t)A(t)}, \end{aligned}$$

3.34 $$\begin{aligned}& C(t) = b(t) \biggl[{\theta_{1}}k_{1}^{{\theta_{1}}-1} \widetilde{f}_{1}\bigl(\alpha (t)\bigr) \biggl(1+f_{2}\bigl( \alpha(t)\bigr) \int_{\alpha(t_{0})}^{\alpha(t)}\widetilde {g}_{1}(\tau) \Delta\tau \biggr) \biggr]\alpha^{\Delta}(t), \end{aligned}$$

3.35 $$\begin{aligned}& \widetilde{f}_{1}(t) = \frac{q_{1}}{l}k_{2}^{q_{1}-l}f_{1}(t), \qquad \widetilde {f}_{3}(t)= \frac{q_{2}}{l}k_{5}^{q_{2}-l}f_{3}(t), \qquad \widetilde{f}_{5}(t)=\frac {q_{3}}{l}k_{8}^{q_{3}-l}f_{5}(t), \end{aligned}$$

3.36 $$\begin{aligned}& \widetilde{g}_{1}(t) = \frac{r_{1}}{q_{1}}k_{2}^{l-q_{1}}k_{3}^{r_{1}-l}g_{1}(t), \qquad \widetilde{g}_{2}(t)=\frac{r_{2}}{q_{2}}k_{5}^{l-q_{2}}k_{6}^{r_{2}-l}g_{2}(t), \end{aligned}$$

3.37 $$\begin{aligned}& \widetilde{g}_{3}(t) = \frac {r_{3}}{q_{3}}k_{8}^{l-q_{3}}k_{9}^{r_{3}-l}g_{3}(t). \end{aligned}$$

### Proof

Denote

3.38 $$\begin{aligned} z(t) =&a(t)+b(t) \int_{\alpha(t_{0})}^{\alpha (t)}f_{1}(s) \biggl[u^{q_{1}}(s)+f_{2}(s) \int_{\alpha(t_{0})}^{s}g_{1}(\tau )u^{r_{1}}(\tau)\Delta\tau \biggr]^{\theta_{1}} \Delta s \\ &{}+ \int_{\beta(t_{0})}^{\beta(T)}f_{3}(s) \biggl[u^{q_{2}}(s)+f_{4}(s) \int_{\beta (t_{0})}^{s}g_{2}(\tau)u^{r_{2}}( \tau)\Delta\tau \biggr]^{\theta_{2}} \Delta s \\ &{}+ \int_{\gamma(t_{0})}^{\gamma(T)}f_{5}(s) \biggl[u^{q_{3}}(s)+f_{6}(s) \int _{\gamma(t_{0})}^{s}g_{3}(\tau)u^{r_{3}}( \tau)\Delta\tau \biggr]^{\theta_{3}} \Delta s,\quad t\in I. \end{aligned}$$

Then *z* is nondecreasing on *I*. From (3.27) and (3.38) we have

3.39 $$ u^{l}(t)\leq z(t), \quad t\in I. $$

Using Lemma 2.6, we obtain

3.40 $$\begin{aligned} z(t) \leq& a(t)+b(t) \int_{\alpha(t_{0})}^{\alpha (t)}f_{1}(s) \biggl[u^{q_{1}}(s)+f_{2}(s) \int_{\alpha(t_{0})}^{s}g_{1}(\tau )u^{r_{1}}(\tau)\Delta\tau \biggr]^{\theta_{1}} \Delta s \\ &{}+ \int_{\beta(t_{0})}^{\beta(T)}f_{3}(s) \biggl[u^{q_{2}}(s)+f_{4}(s) \int_{\beta (t_{0})}^{s}g_{2}(\tau)u^{r_{2}}( \tau)\Delta\tau \biggr]^{\theta_{2}} \Delta s \\ &{}+ \int_{\gamma(t_{0})}^{\gamma(T)}f_{5}(s) \biggl[u^{q_{3}}(s)+f_{6}(s) \int _{\gamma(t_{0})}^{s}g_{3}(\tau)u^{r_{3}}( \tau)\Delta\tau \biggr]^{\theta_{3}} \Delta s \\ \leq&a(t)+b(t) \int_{\alpha(t_{0})}^{\alpha(t)} \biggl\{ {\theta _{1}}k_{1}^{{\theta_{1}}-1}f_{1}(s) \biggl[\frac{q_{1}}{l}k_{2}^{q_{1}-l}u^{l}(s)+ \frac {l-q_{1}}{l}k_{2}^{q_{1}} \\ &{}+f_{2}(s) \int_{\alpha(t_{0})}^{s}g_{1}(\tau) \biggl( \frac {r_{1}}{l}k_{3}^{r_{1}-l}u^{l}(\tau)+ \frac{l-r_{1}}{l}k_{3}^{r_{1}} \biggr) \Delta\tau \biggr]+(1-{ \theta_{1}})k_{1}^{\theta_{1}}f_{1}(s) \biggr\} \Delta s \\ &{}+ \int_{\beta(t_{0})}^{\beta(T)} \biggl\{ {\theta_{2}}k_{4}^{{\theta _{2}}-1}f_{3}(s) \biggl[\frac{q_{2}}{l}k_{5}^{q_{2}-l}u^{l}(s)+ \frac {l-q_{2}}{l}k_{5}^{q_{2}} \\ &{}+f_{4}(s) \int_{\beta(t_{0})}^{s}g_{2}(\tau) \biggl( \frac {r_{2}}{l}k_{6}^{r_{2}-l}u^{l}(\tau)+ \frac{l-r_{2}}{l}k_{6}^{r_{2}} \biggr) \Delta\tau \biggr]+(1-{ \theta_{2}})k_{4}^{\theta_{2}}f_{3}(s) \biggr\} \Delta s \\ &{}+ \int_{\gamma(t_{0})}^{\gamma(T)} \biggl\{ {\theta_{3}}k_{7}^{{\theta _{3}}-1}f_{5}(s) \biggl[\frac{q_{3}}{l}k_{8}^{q_{3}-l}u^{l}(s)+ \frac {l-q_{3}}{l}k_{8}^{q_{3}} \\ &{}+f_{6}(s) \int_{\gamma(t_{0})}^{s}g_{3}(\tau) \biggl( \frac {r_{3}}{l}k_{9}^{r_{3}-l}u^{l}(\tau)+ \frac{l-r_{3}}{l}k_{9}^{r_{3}} \biggr)\Delta\tau \biggr]+(1-{ \theta_{3}})k_{7}^{\theta_{3}}f_{5}(s) \biggr\} \Delta s. \end{aligned}$$

Using (3.30), (3.31), (3.35)–(3.37), and (3.39), we get

3.41 $$\begin{aligned} z(t) \leq& \widetilde{K}+\widetilde{V}(t)+b(t) \int _{\alpha(t_{0})}^{\alpha(t)} \biggl\{ {\theta_{1}}k_{1}^{{\theta_{1}}-1} \widetilde {f}_{1}(s) \biggl[z(s) +f_{2}(s) \int_{\alpha(t_{0})}^{s}\widetilde{g}_{1}(\tau)z( \tau) \Delta\tau \biggr] \biggr\} \Delta s \\ &{}+ \int_{\beta(t_{0})}^{\beta(T)} \biggl\{ {\theta_{2}}k_{4}^{{\theta _{2}}-1} \widetilde{f}_{3}(s) \biggl[z(s) +f_{4}(s) \int_{\beta(t_{0})}^{s}\widetilde{g}_{2}(\tau)z( \tau) \Delta\tau \biggr] \biggr\} \Delta s \\ &{}+ \int_{\gamma(t_{0})}^{\gamma(T)} \biggl\{ {\theta_{3}}k_{7}^{{\theta _{3}}-1} \widetilde{f}_{5}(s) \biggl[z(s) +f_{6}(s) \int_{\gamma(t_{0})}^{s}\widetilde{g}_{3}(\tau)z( \tau)\Delta\tau \biggr] \biggr\} \Delta s,\quad t\in I. \end{aligned}$$

It is similar to the proof of Theorem 3.1, we get

$$z(t)\leq\frac{\widetilde{K}+\widetilde{V}(T)}{1-\lambda}e_{B \oplus C}(t,t_{0}),\quad t\in I. $$

Then, using $u^{l}(t)\leq z(t)$, we have (3.29). This completes the proof. □

### Remark 3.1

If we take $a(t)\equiv u_{0}$, $b(t)\equiv1$, $\alpha (t)\equiv t$, $\beta(t)\equiv t$, $f_{2}(t)\equiv1$, and $f_{4}(t)=f_{5}(t)\equiv0$, then Corollary 3.1 reduces to [22, Theorem 2.2]. If we take $a(t)\equiv k$, $b(t)\equiv1$, $\alpha(t)\equiv t$, $\beta (t)\equiv t$, $p=1$, $q_{1}=r_{1}=q_{2}=r_{2}$, $\theta_{1}=\theta_{2}=1$, $f_{1}(t)=f_{3}(t)$, $f_{2}(t)=f_{4}(t)$, $g_{1}(t)=g_{2}(t)$, and $f_{5}(t)\equiv0$, then Theorem 3.2 gives an exact estimation for the solution of (3.27) compared with the result of [27, Theorem 4].

### Theorem 3.3

*Assume that* ($\mathrm{H}_{1}$)*–*($\mathrm{H}_{3}$), ($\mathrm{H}_{5}$), ($\mathrm{H}_{6}$) *hold*, $0\leq p\leq1$, $0\leq q\leq1$ *are constants*, $\mu(t)\widetilde{A}(t)<1$, $v_{t}^{\Delta}(t,s)\geq0$, $w_{t}^{\Delta}(t,s)\geq0$ *for* $t\geq s$ *and* (2.3) *holds*. *Suppose that* *u* *satisfies*

3.42 $$\begin{aligned} u(t) \leq& a(t)+b(t) \int_{t_{0}}^{t} \biggl[v(t,s)u(s)+w(t,s) \int_{\alpha(t_{0})}^{\alpha(s)}f(\tau)u(\tau)\Delta \tau \biggr]^{p} \Delta s \\ &{}+ \int_{t_{0}}^{T} \biggl[m(T,s)u(s)+n(T,s) \int_{\beta(t_{0})}^{\beta (s)}g(\tau)u(\tau)\Delta\tau \biggr]^{q} \Delta s, \quad t\in I. \end{aligned}$$

*If there exist positive constants* $k_{i}$ ($i=1,2$) *such that*

3.43 $$\begin{aligned} \lambda :=& \int_{\beta(t_{0})}^{\beta(T)} \biggl[qk_{2}^{q-1}f_{3}(s) \biggl(e_{B \oplus C}(s,t_{0})+f_{4}(s) \int_{\beta (t_{0})}^{s}g_{2}(\tau)e_{B \oplus C}( \tau,t_{0})\Delta\tau \biggr) \biggr] \Delta s \\ &{}+ \int_{\gamma(t_{0})}^{\gamma(T)} \biggl[rk_{3}^{r-1}f_{5}(s) \biggl(e_{B \oplus C}(s,t_{0})+f_{6}(s) \int_{\gamma(t_{0})}^{s}g_{3}(\tau)e_{B \oplus C}( \tau ,t_{0})\Delta\tau \biggr) \biggr] \Delta s< 1, \end{aligned}$$

*then*

3.44 $$ u(t) \leq \frac{K+V(T)}{1-\lambda}e_{B \oplus C}(t,t_{0}),\quad t\in I, $$

*where*

3.45 $$\begin{aligned}& K= \int_{t_{0}}^{T}(1-q)k_{2}^{q} \Delta s, \end{aligned}$$

3.46 $$\begin{aligned}& V(t)=a(t)+b(t) \int_{t_{0}}^{t}(1-p)k_{1}^{p} \Delta s, \end{aligned}$$

3.47 $$\begin{aligned}& \begin{aligned} &\widetilde{A}(t)=b^{\Delta}(t) \int_{t_{0}}^{\sigma(t)} \biggl[v\bigl(\sigma(t),s\bigr)+w \bigl(\sigma(t),s\bigr) \int_{\alpha(t_{0})}^{\alpha(s)}g(\tau )\Delta\tau \biggr] \Delta s, \\ &\widetilde{B}(t)=\frac{\widetilde{A}(t)}{1-\mu(t)\widetilde {A}(t)}, \end{aligned} \end{aligned}$$

3.48 $$\begin{aligned}& \begin{aligned}[b] \widetilde{C}(t)&=b(t) \biggl[v\bigl(\sigma(t),t\bigr)+w \bigl(\sigma (t),t\bigr) \int_{\alpha(t_{0})}^{\alpha(t)}g(\tau)\Delta\tau \\ &\quad {}+ \int_{t_{0}}^{t} \biggl[v^{\Delta}_{t}(t,s)+w^{\Delta}_{t}(t,s) \int_{\alpha(t_{0})}^{\alpha(s)}g(\tau )\Delta\tau \biggr]\Delta s \biggr]. \end{aligned} \end{aligned}$$

### Proof

Denote

3.49 $$\begin{aligned} z(t) =&a(t)+ b(t) \int_{t_{0}}^{t} \biggl[v(t,s)u(s)+w(t,s) \int_{\alpha(t_{0})}^{\alpha(s)}f(\tau)u(\tau)\Delta \tau \biggr]^{p} \Delta s \\ &{}+ \int_{t_{0}}^{T} \biggl[m(T,s)u(s)+n(T,s) \int_{\beta(t_{0})}^{\beta (s)}g(\tau)u(\tau)\Delta\tau \biggr]^{q} \Delta s, \quad t\in I. \end{aligned}$$

Then *z* is nondecreasing on *I*. From (3.42) and (3.49), we have

3.50 $$ u(t)\leq z(t),\quad t\in I. $$

Now, using Lemma 2.6 for $a=v(t,s)u(s)+w(t,s)\int_{\alpha(t_{0})}^{\alpha (s)}f(\tau)u(\tau)\Delta\tau$, and $m(T,s)u(s)+n(T,s)\int_{\beta(t_{0})}^{\beta(s)}g(\tau)u(\tau)\Delta \tau$ with $m=1$ and $n=p, q$ for any $k_{1}, k_{2}>0$, respectively, we have

3.51 $$\begin{aligned} z(t) \leq& a(t)+b(t) \int_{t_{0}}^{t} \biggl\{ pk_{1}^{p-1} \biggl[v(t,s)u(s)+w(t,s) \int_{\alpha(t_{0})}^{\alpha(s)}f(\tau )u(\tau)\Delta\tau \biggr]+(1-p)k_{1}^{p} \biggr\} \Delta s \\ &{}+ \int_{t_{0}}^{T} \biggl\{ qk_{2}^{q-1} \biggl[m(T,s)u(s)+n(T,s) \int_{\beta (t_{0})}^{\beta(s)}g(\tau)u(\tau)\Delta\tau \biggr] \\ &{}+(1-q)k_{2}^{q} \biggr\} \Delta s,\quad t\in I. \end{aligned}$$

Now, using (3.45) and (3.46) and (3.51), we have

3.52 $$\begin{aligned} z(t) \leq& K+V(t)+b(t) \int_{t_{0}}^{t} \biggl\{ pk_{1}^{p-1} \biggl[v(t,s)z(s)+w(t,s) \int_{\alpha(t_{0})}^{\alpha(s)}f(\tau )z(\tau)\Delta\tau \biggr] \biggr\} \Delta s \\ &{}+ \int_{t_{0}}^{T} \biggl\{ qk_{2}^{q-1} \biggl[m(T,s)z(s)+n(T,s) \int_{\beta (t_{0})}^{\beta(s)}g(\tau)z(\tau)\Delta\tau \biggr] \biggr\} \Delta s, \quad t\in I. \end{aligned}$$

Since $V(t)$ is nondecreasing on *I*, then for $t\in I$, from the above inequality we have

3.53 $$\begin{aligned} z(t) \leq& K+V(T)+b(t) \int_{t_{0}}^{t} \biggl\{ pk_{1}^{p-1} \biggl[v(t,s)z(s)+w(t,s) \int _{\alpha(t_{0})}^{\alpha(s)}f(\tau)z(\tau)\Delta\tau \biggr] \biggr\} \Delta s \\ &{}+ \int_{t_{0}}^{T} \biggl\{ qk_{2}^{q-1} \biggl[m(T,s)z(s)+n(T,s) \int_{\beta (t_{0})}^{\beta(s)}g(\tau)z(\tau)\Delta\tau \biggr] \biggr\} \Delta s,\quad t\in I. \end{aligned}$$

Let

3.54 $$ M=K+V(T)+ \int_{t_{0}}^{T} \biggl\{ qk_{2}^{q-1} \biggl[m(T,s)z(s)+n(T,s) \int _{\beta(t_{0})}^{\beta(s)}g(\tau)z(\tau)\Delta\tau \biggr] \biggr\} \Delta s. $$

Then (3.53) can be restated as

3.55 $$ z(t)\leq M+b(t) \int_{t_{0}}^{t} \biggl\{ pk_{1}^{p-1} \biggl[v(t,s)z(s)+w(t,s) \int _{\alpha(t_{0})}^{\alpha(s)}f(\tau)z(\tau)\Delta\tau \biggr] \biggr\} \Delta s, \quad t\in I. $$

Set

3.56 $$ y(t)=M+b(t) \int_{t_{0}}^{t} \biggl\{ pk_{1}^{p-1} \biggl[v(t,s)z(s)+w(t,s) \int _{\alpha(t_{0})}^{\alpha(s)}f(\tau)z(\tau)\Delta\tau \biggr] \biggr\} \Delta s,\quad t\in I. $$

Then $y(t)$ is nondecreasing, and from (3.55) and (3.56) we obtain

3.57 $$ z(t)\leq y(t), \quad t\in I. $$

Using Lemma 2.3, taking the delta derivative of (3.56), and from (3.57), we have

3.58 $$\begin{aligned} y^{\Delta}(t) =&b^{\Delta}(t) \int_{t_{0}}^{\sigma(t)} \biggl\{ pk_{1}^{p-1} \biggl[v\bigl(\sigma(t),s\bigr)z(s)+w\bigl(\sigma(t),s\bigr) \int_{\alpha(t_{0})}^{\alpha (s)}f(\tau)z(\tau)\Delta\tau \biggr] \biggr\} \Delta s \\ &{}+b(t) \biggl\{ pk_{1}^{p-1} \biggl[v\bigl(\sigma(t),t \bigr)z(t)+w\bigl(\sigma(t),t\bigr) \int _{\alpha(t_{0})}^{\alpha(t)}f(\tau)z(\tau)\Delta\tau \biggr] \biggr\} \\ &{}+b(t) \int_{t_{0}}^{t} \biggl\{ pk_{1}^{p-1} \biggl[v_{t}^{\Delta}(t,s)z(s)+w_{t}^{\Delta}(t,s) \int_{\alpha(t_{0})}^{\alpha(s)}f(\tau)z(\tau )\Delta\tau \biggr] \biggr\} \Delta s \\ \ \leq&b^{\Delta}(t) \int_{t_{0}}^{\sigma(t)} \biggl\{ pk_{1}^{p-1} \biggl[v\bigl(\sigma (t),s\bigr)y(s)+w\bigl(\sigma(t),s\bigr) \int_{\alpha(t_{0})}^{\alpha(s)}f(\tau)y(\tau )\Delta\tau \biggr] \biggr\} \Delta s \\ &{}+b(t) \biggl\{ pk_{1}^{p-1} \biggl[v\bigl(\sigma(t),t \bigr)y(t)+w\bigl(\sigma(t),t\bigr) \int _{\alpha(t_{0})}^{\alpha(t)}f(\tau)y(\tau)\Delta\tau \biggr] \biggr\} \\ &{}+b(t) \int_{t_{0}}^{t} \biggl\{ pk_{1}^{p-1} \biggl[v_{t}^{\Delta}(t,s)y(s)+w_{t}^{\Delta}(t,s) \int_{\alpha(t_{0})}^{\alpha(s)}f(\tau)y(\tau )\Delta\tau \biggr] \biggr\} \Delta s \\ \ \leq&y\bigl(\sigma(t)\bigr)b^{\Delta}(t) \int_{t_{0}}^{\sigma(t)} \biggl\{ pk_{1}^{p-1} \biggl[v\bigl(\sigma(t),s\bigr)+w\bigl(\sigma(t),s\bigr) \int_{\alpha(t_{0})}^{\alpha(s)}f(\tau )\Delta\tau \biggr] \biggr\} \Delta s \\ &{}+b(t)y(t) \biggl\{ pk_{1}^{p-1} \biggl[v\bigl(\sigma(t),t \bigr)+w\bigl(\sigma(t),t\bigr) \int _{\alpha(t_{0})}^{\alpha(t)}f(\tau)\Delta\tau \biggr] \\ &{}+ \int_{t_{0}}^{t}pk_{1}^{p-1} \biggl[v_{t}^{\Delta}(t,s)+w_{t}^{\Delta}(t,s) \int _{\alpha(t_{0})}^{\alpha(s)}f(\tau)\Delta\tau \biggr]\Delta s \biggr\} \\ =&\widetilde{A}(t)y\bigl(\sigma(t)\bigr)+\widetilde{C}(t)y(t), \quad t\in I, \end{aligned}$$

where $\widetilde{A}(t)$ and $\widetilde{C}(t)$ are defined as in (3.47) and (3.48). It is similar to the proof of Theorem 3.1, we get (3.44). This completes the proof. □

### Theorem 3.4

*Assume that* ($\mathrm{H}_{3}$)*–*($\mathrm{H}_{5}$) *hold*, $0\leq q_{i}\leq l$, $0\leq r_{i}\leq l$, $l\neq 0$, $0\leq\theta_{i}\leq1$ ($i=1,2$) *are constants*, *and* $\mu(t)A(t)<1$, $v_{t}^{\Delta}(t,s)\geq0$, $w_{t}^{\Delta}(t,s)\geq0$ *for* $t\geq s$. *And assume* (2.3) *holds*. *Suppose that* *u* *satisfies*

3.59 $$\begin{aligned} \begin{aligned}[b] u^{l}(t) &\leq a(t)+ \int_{t_{0}}^{t}f_{1}(s) \biggl[u^{q_{1}}(s)+ \int_{t_{0}}^{s}v(s,\tau)u^{r_{1}}(\tau)\Delta \tau \biggr]^{\theta_{1}} \Delta s \\ &\quad {}+ \int_{t_{0}}^{T}f_{2}(s) \biggl[u^{q_{2}}(s)+ \int_{t_{0}}^{s}w(s,\tau )u^{r_{2}}(\tau)\Delta \tau \biggr]^{\theta_{2}} \Delta s,\quad t\in I. \end{aligned} \end{aligned}$$

*If there exist positive constants* $k_{i}$ ($i=1,2,3,4,5,6$) *such that*

3.60 $$\begin{aligned} \lambda :=& \int_{t_{0}}^{T}\widetilde{f}_{2}(s) \biggl[1+ \int_{t_{0}}^{s}\widetilde {f}_{1}(s)e_{A}(s,t_{0}) \Delta s \\ &{}+ \int_{t_{0}}^{s}\widetilde{w}(s,\tau) \biggl(1+ \int_{t_{0}}^{\tau}\widetilde {f}_{1}( \xi)e_{A}(\xi,t_{0})\Delta\xi \biggr) \Delta\tau \biggr] \Delta s< 1, \end{aligned}$$

*then*

3.61 $$ u(t) \leq \biggl[\frac{K+F(t)}{1-\lambda} \biggl(1+ \int_{t_{0}}^{t}\widetilde {f}_{1}(s)e_{A}(s,t_{0}) \Delta s \biggr) \biggr]^{\frac{1}{l}},\quad t\in I, $$

*where*

3.62 $$\begin{aligned}& \begin{aligned}[b] K&= \int_{t_{0}}^{T} \biggl\{ {\theta_{2}}k_{4}^{{\theta _{2}}-1}f_{2}(s) \biggl[\frac{l-q_{2}}{l}k_{5}^{q_{2}} + \int_{t_{0}}^{s}w(s,\tau) \biggl(\frac{l-r_{2}}{l}k_{6}^{r_{2}} \biggr)\Delta\tau \biggr] \\ &\quad {}+(1-{\theta_{2}})k_{4}^{\theta_{1}}f_{2}(s) \biggr\} \Delta s, \end{aligned} \end{aligned}$$

3.63 $$\begin{aligned}& \begin{aligned}[b] F(t)&=a(t)+ \int_{t_{0}}^{t} \biggl\{ {\theta_{1}}k_{1}^{{\theta _{1}}-1}f_{1}(s) \biggl[\frac{l-q_{1}}{l}k_{2}^{q_{1}} + \int_{t_{0}}^{s}v(s,\tau) \biggl(\frac{l-r_{1}}{l}k_{3}^{r_{1}} \biggr)\Delta\tau \biggr] \\ &\quad {}+(1-{\theta_{1}})k_{1}^{\theta_{1}}f_{1}(s) \biggr\} \Delta s, \end{aligned} \end{aligned}$$

3.64 $$\begin{aligned}& A(t)=\widetilde{f}_{1}(t)+\widetilde{v}\bigl(\sigma(t),t\bigr)+ \int _{t_{0}}^{t}\widetilde{v}^{\Delta}_{t}(t, \tau)\Delta\tau, \end{aligned}$$

3.65 $$\begin{aligned}& \widetilde{f}_{1}(t)=\frac{q_{1}}{l}{\theta_{1}}k_{1}^{{\theta _{1}}-1}k_{2}^{q_{1}-l}f_{1}(t),\qquad \widetilde{f}_{2}(t)=\frac{q_{2}}{l}{\theta _{2}}k_{4}^{{\theta_{2}}-1}k_{5}^{q_{2}-l}f_{2}(t), \end{aligned}$$

3.66 $$\begin{aligned}& \widetilde{v}(s,t)=\frac{r_{1}}{q_{1}}k_{2}^{l-q_{1}}k_{3}^{r_{1}-l}v(s,t),\qquad \widetilde{w}(s,t)=\frac{r_{2}}{q_{2}}k_{5}^{l-q_{2}}k_{6}^{r_{2}-l}w(s,t). \end{aligned}$$

### Proof

Denote

3.67 $$\begin{aligned} z(t) =& a(t)+ \int_{t_{0}}^{t}f_{1}(s) \biggl[u^{q_{1}}(s)+ \int _{t_{0}}^{s}v(s,\tau)u^{r_{1}}(\tau)\Delta \tau \biggr]^{\theta_{1}} \Delta s \\ &{}+ \int_{t_{0}}^{T}f_{2}(s) \biggl[u^{q_{2}}(s)+ \int_{t_{0}}^{s}w(s,\tau )u^{r_{2}}(\tau)\Delta \tau \biggr]^{\theta_{2}} \Delta s, \quad t\in I. \end{aligned}$$

Then *z* is nondecreasing on *I*. From (3.59) and (3.67) we have

3.68 $$ u^{l}(t)\leq z(t),\quad t\in I. $$

Using Lemma 2.6, we obtain

3.69 $$\begin{aligned} z(t) \leq&a(t)+ \int_{t_{0}}^{t} \biggl\{ {\theta _{1}}k_{1}^{{\theta_{1}}-1}f_{1}(s) \biggl[\frac{q_{1}}{l}k_{2}^{q_{1}-l}u^{l}(s)+ \frac {l-q_{1}}{l}k_{2}^{q_{1}} \\ &{}+ \int_{t_{0}}^{s}v(s,\tau) \biggl(\frac{r_{1}}{l}k_{3}^{r_{1}-l}u^{l}( \tau)+\frac {l-r_{1}}{l}k_{3}^{r_{1}} \biggr)\Delta\tau \biggr] +(1-{\theta_{1}})k_{1}^{\theta_{1}}f_{1}(s) \biggr\} \Delta s \\ &{}+ \int_{t_{0}}^{T} \biggl\{ {\theta_{2}}k_{4}^{{\theta_{2}}-1}f_{2}(s) \biggl[\frac {q_{2}}{l}k_{5}^{q_{2}-l}u^{l}(s)+ \frac{l-q_{2}}{l}k_{5}^{q_{2}} \\ &{}+ \int_{t_{0}}^{s}w(s,\tau) \biggl(\frac{r_{2}}{l}k_{6}^{r_{2}-l}u^{l}( \tau)+\frac {l-r_{2}}{l}k_{6}^{r_{2}} \biggr)\Delta\tau \biggr] \\ &{}+(1-{\theta_{2}})k_{4}^{\theta_{1}}f_{2}(s) \biggr\} \Delta s, \quad t\in I. \end{aligned}$$

Using (3.62), (3.63), (3.65), (3.66), (3.68), and (3.69), we get

3.70 $$\begin{aligned} z(t) \leq& K+F(t)+ \int_{t_{0}}^{t}\widetilde{f}_{1}(s) \biggl[z(s) + \int_{t_{0}}^{s}\widetilde{v}(s,\tau)z(\tau) \Delta\tau \biggr] \Delta s \\ &{}+ \int_{t_{0}}^{T}\widetilde{f}_{2}(s) \biggl[z(s) + \int_{t_{0}}^{s}\widetilde{w}(s,\tau)z(\tau) \Delta\tau \biggr] \Delta s,\quad t\in I. \end{aligned}$$

Since $F(t)$ is nondecreasing on *I*, then for $t\in I$, from the above inequality we have

3.71 $$\begin{aligned} z(t) \leq& K+F(T)+ \int_{t_{0}}^{t}\widetilde{f}_{1}(s) \biggl[z(s) + \int_{t_{0}}^{s}\widetilde{v}(s,\tau)z(\tau) \Delta\tau \biggr] \Delta s \\ &{}+ \int_{t_{0}}^{T}\widetilde{f}_{2}(s) \biggl[z(s) + \int_{t_{0}}^{s}\widetilde{w}(s,\tau)z(\tau) \Delta\tau \biggr] \Delta s,\quad t\in I. \end{aligned}$$

Let

3.72 $$ M = K+F(T)+ \int_{t_{0}}^{T}\widetilde{f}_{2}(s) \biggl[z(s) + \int_{t_{0}}^{s}\widetilde{w}(s,\tau)z(\tau) \Delta\tau \biggr] \Delta s. $$

Then (3.71) can be restated as

3.73 $$ z(t)\leq M+ \int_{t_{0}}^{t}\widetilde{f}_{1}(s) \biggl[z(s) + \int_{t_{0}}^{s}\widetilde{v}(s,\tau)z(\tau) \Delta\tau \biggr] \Delta s,\quad t\in I. $$

Set

3.74 $$ N(t)=M+ \int_{t_{0}}^{t}\widetilde{f}_{1}(s) \biggl[z(s) + \int_{t_{0}}^{s}\widetilde{v}(s,\tau)z(\tau) \Delta\tau \biggr] \Delta s,\quad t\in I. $$

Then $N(t)$ is nondecreasing, and from (3.73) and (3.74) we obtain

3.75 $$ z(t)\leq N(t),\quad t\in I. $$

Taking the delta derivative of (3.74) and from (3.75), we get

3.76 $$\begin{aligned} \begin{aligned}[b] N^{\Delta}(t)&=\widetilde{f}_{1}(t)z(t)+\widetilde {f}_{1}(t) \int_{t_{0}}^{t}\widetilde{v}(t,\tau)z(\tau)\Delta\tau \\ &\leq\widetilde{f}_{1}(t) \biggl[N(t)+ \int_{t_{0}}^{t}\widetilde{v}(t,\tau )N(\tau)\Delta\tau \biggr], \quad t\in I. \end{aligned} \end{aligned}$$

Let

3.77 $$ V(t)=N(t)+ \int_{t_{0}}^{t}\widetilde{v}(t,\tau)N(\tau)\Delta\tau, \quad t\in I. $$

Obviously,

3.78 $$ V(t_{0})=N(t_{0}),\qquad N(t)\leq V(t),\qquad N^{\Delta}(t)\leq\widetilde {f}_{1}(t)V(t). $$

From Lemma 2.4, (3.77), and (3.78), we obtain

$$\begin{aligned} V^{\Delta}(t) =&N^{\Delta}(t)+\widetilde{v}\bigl(\sigma (t),t \bigr)N(t)+ \int_{t_{0}}^{t}\widetilde{v}^{\Delta}_{t}(t, \tau)N(\tau)\Delta \tau \\ \leq& \biggl[\widetilde{f}_{1}(t)+\widetilde{v}\bigl(\sigma(t),t \bigr)+ \int _{t_{0}}^{t}\widetilde{v}^{\Delta}_{t}(t, \tau)\Delta\tau \biggr]V(t) \\ =&A(t)V(t), \quad t\in I. \end{aligned}$$

It is easy to see that $A\in\mathcal{R}^{+}$. Therefore, from Lemma 2.5 and the above inequality, we have

3.79 $$ V(t)\leq V(t_{0})e_{\widetilde{A}}(t,t_{0})=N(t_{0})e_{A}(t,t_{0}), \quad t\in I. $$

Combining (3.78) and (3.79), we get

3.80 $$ N^{\Delta}(t)\leq\widetilde{f}_{1}(t)N(t_{0})e_{A}(t,t_{0}). $$

Setting $t=\tau$ in (3.80), integrating it from $t_{0}$ to *t*, we easily obtain

3.81 $$ N(t)\leq N(t_{0})+N(t_{0}) \int_{t_{0}}^{t}\widetilde {f}_{1}(s)e_{A}(s,t_{0}) \Delta s, \quad t\in I. $$

By (3.74) and (3.81), we get

3.82 $$ N(t)\leq M \biggl(1+ \int_{t_{0}}^{t}\widetilde{f}_{1}(s)e_{A}(s,t_{0}) \Delta s \biggr),\quad t\in I. $$

From (3.75) and (3.82), we have

3.83 $$ z(t)\leq M \biggl(1+ \int_{t_{0}}^{t}\widetilde{f}_{1}(s)e_{A}(s,t_{0}) \Delta s \biggr),\quad t\in I. $$

Using (3.83) on the right-hand side of (3.72) and according to (3.60), we obtain

3.84 $$ M\leq\frac{K+F(T)}{1-\lambda}. $$

From (3.83) and (3.84), we obtain

3.85 $$ z(t)\leq\frac{K+F(T)}{1-\lambda} \biggl(1+ \int_{t_{0}}^{t}\widetilde {f}_{1}(s)e_{A}(s,t_{0}) \Delta s \biggr),\quad t\in I. $$

Then using (3.68), we have (3.61). This completes the proof. □

### Remark 3.2

If we take $l=q_{1}=r_{1}=\theta_{1}=1$, and $f_{2}(t)\equiv 0$, then Theorem 3.4 reduces to [28, Theorem 3.2].

## Applications

In this section, we will present some simple applications for our results. First, we consider the following Volterra–Fredholm type dynamic integral equation:

4.1 $$\begin{aligned} u(t) =& K_{0}+ \int_{1}^{t}\frac{2}{s} \biggl[u(s)+ \frac {s^{2}}{s+1} \int_{1}^{s}\frac{1+q}{q^{2}\tau^{3}}u(\tau)\Delta\tau \biggr]^{\frac{1}{2}} \Delta s \\ &{}+ \int_{1}^{T}\frac{3}{2s} \biggl[u(s)+ \frac{s^{2}}{s+1} \int_{1}^{s}\frac {1+q}{q^{2}\tau^{3}}u(\tau)\Delta\tau \biggr]^{\frac{1}{3}} \Delta s, \quad t\in I, \end{aligned}$$

on time scales $\mathbb{T}=q^{\mathbb{N}_{0}}$, where $q^{\mathbb{N}_{0}}=\{ q^{n}: n\in\mathbb{N}_{0}, q>1\}$, and $I=[1,T]\cap q^{\mathbb{N}_{0}}$, $T=q^{N}$, *N* is some positive integer and $K_{0}\in\mathbb{R}$.

The following theorem gives the bound on the solution of Eq. (4.1).

### Theorem 4.1

*Suppose that* *u* *is a solution of Eq*. (4.1) *on* *I*. *If there exist positive constants* $k_{1}$ *and* $k_{2}$ *such that*

4.2 $$\begin{aligned} \lambda :=& \int_{1}^{T} \biggl\{ k_{2}^{-\frac{2}{3}} \frac{1}{2s} \biggl[\prod_{\tau\in [1,s)}\biggl[1+(q-1) \frac{1}{2}k_{1}^{-\frac{1}{2}}\tau\biggr] \\ &{}+\frac{s^{2}}{s+1} \int_{1}^{s}\frac{1+q}{q^{2}\tau^{3}}\prod _{\nu\in[1,\tau )}\biggl[1+(q-1)\frac{1}{2}k_{1}^{-\frac{1}{2}} \nu\biggr]\Delta\tau \biggr] \biggr\} \Delta s< 1, \end{aligned}$$

*then*

4.3 $$ \bigl\vert u(t) \bigr\vert \leq \frac{(\frac{\ln T}{\ln q}-1)(q-1)(k_{2}^{\frac {1}{3}}+k_{1}^{\frac{1}{2}})+|K_{0}|}{1-\lambda}\prod _{s\in [1,t)}\biggl[1+(q-1)\frac{1}{2}k_{1}^{-\frac{1}{2}}s \biggr], \quad t\in I. $$

### Proof

From (4.1), we get

4.4 $$\begin{aligned} \bigl\vert u(t) \bigr\vert =& \vert K_{0} \vert + \int_{1}^{t}\frac{2}{s} \biggl[ \bigl\vert u(s) \bigr\vert +\frac{s^{2}}{s+1} \int_{1}^{s}\frac{1+q}{q^{2}\tau^{3}} \bigl\vert u( \tau) \bigr\vert \Delta \tau \biggr]^{\frac{1}{2}} \Delta s \\ &{}+ \int_{1}^{T}\frac{3}{2s} \biggl[ \bigl\vert u(s) \bigr\vert +\frac{s^{2}}{s+1} \int_{1}^{s}\frac {1+q}{q^{2}\tau^{3}} \bigl\vert u( \tau) \bigr\vert \Delta\tau \biggr]^{\frac{1}{3}} \Delta s,\quad t\in I. \end{aligned}$$

Take $t_{0}=1$, $a(t)=|K_{0}|$, $b(t)=1$, $f_{1}(t)=\frac{2}{t}$, $f_{2}(t)=f_{4}(t)=\frac{t^{2}}{t+1}$, $f_{3}(t)=\frac{3}{2t}$, $f_{5}(t)=f_{6}(t)\equiv0$, $g_{1}(t)=g_{2}(t)=\frac{1+q}{q^{2}t^{3}}$, $\alpha (t)=t$, $\beta(t)=t$, $p=\frac{1}{2}$, and $q=\frac{1}{3}$ in Theorem 3.1, on the basis of a straightforward computation, we have

$$\begin{aligned}& K = \int_{1}^{T}\frac{2}{3}k_{2}^{\frac{1}{3}} \frac{3}{2s} \Delta s= \biggl(\frac{\ln T}{\ln q}-1 \biggr) (q-1)k_{2}^{\frac{1}{3}}, \\& V(t) = |K_{0}|+ \int_{1}^{t}\frac{1}{2}k_{1}^{\frac{1}{2}} \frac{2}{s} \Delta s=|K_{0}|+ \biggl(\frac{\ln t}{\ln q}-1 \biggr) (q-1)k_{1}^{\frac{1}{2}}, \\& A(t) = 0, \qquad B(t)=0,\qquad C(t)=\frac{1}{2}k_{1}^{-\frac{1}{2}}, \\& e_{B \oplus C}(t,1) = \prod_{s\in[1,t)}\biggl[1+(q-1) \frac{1}{2}k_{1}^{-\frac {1}{2}}s\biggr]. \end{aligned}$$

Using Theorem 3.1, we obtain the desired inequality (4.3). □

Secondly, we consider the following retarded Volterra–Fredholm type dynamic integral equation on $\mathbb{R}$:

4.5 $$\begin{aligned} u(t) =& K_{0}+ \int_{0}^{\frac{t}{2}}\frac{3s}{1+3s} \biggl[u(s)+ \frac{1}{s^{4}} \int_{0}^{s}\tau^{2}u(\tau)\,d\tau \biggr]^{\frac{1}{2}} \,ds \\ &{}+ \int_{0}^{\frac{T}{3}}e^{-s} \biggl[u(s)+ \int_{0}^{s}u(\tau)\,d\tau \biggr]^{\frac{1}{3}} \,ds, \quad t\in I, \end{aligned}$$

where $I=[0,T]$, *T* is some positive real number and $K_{0}\in\mathbb{R}$.

The next result also deals with the boundedness of the solutions of Eq. (4.5).

### Theorem 4.2

*Suppose that* *u* *is a solution of Eq*. (4.5) *on* *I*. *If there exist positive constants* $k_{1}$ *and* $k_{2}$ *such that*

4.6 $$ \lambda:=\frac{1}{3}k_{2}^{-\frac{2}{3}} \biggl[ \frac {4k_{1}^{\frac{1}{2}}+1}{\frac{1}{4}k_{1}^{-\frac{1}{2}}-1} \exp \biggl\{ \biggl(\frac{1}{4}k_{1}^{-\frac{1}{2}}-1 \biggr)\frac{T}{3} \biggr\} +4k_{1}^{\frac{1}{2}}e^{-\frac{T}{3}}- \frac{4k_{1}^{\frac{1}{2}}+1}{\frac {1}{4}k_{1}^{-\frac{1}{2}}-1} - 4k_{1}^{\frac{1}{2}} \biggr] < 1, $$

*then*

4.7 $$ \bigl\vert u(t) \bigr\vert \leq\frac{\frac{2}{3}k_{2}^{\frac{1}{3}} (1-e^{-\frac{T}{3}} ) +|K_{0}|+\frac{1}{2}k_{1}^{\frac{1}{2}} (\frac{T}{2}-\frac{1}{3}\ln (1+\frac{3T}{2}) )}{1-\lambda}\exp \biggl\{ \frac{1}{4}k_{1}^{-\frac {1}{2}}s \biggr\} ,\quad t\in I. $$

### Proof

From (4.5), we have

4.8 $$\begin{aligned} \bigl|u(t)\bigr| \leq&|K_{0}|+ \int_{0}^{\frac{t}{2}}\frac {3s}{1+3s} \biggl[\bigl|u(s)\bigr|+ \frac{1}{s^{4}} \int_{0}^{s}\tau^{2}\bigl|u(\tau)\bigr|\,d\tau \biggr]^{\frac{1}{2}} \,ds \\ &{}+ \int_{0}^{\frac{T}{3}}e^{-s} \biggl[\bigl|u(s)\bigr|+ \int_{0}^{s}\bigl|u(\tau)\bigr|\,d\tau \biggr]^{\frac{1}{3}} \,ds,\quad t\in I. \end{aligned}$$

Take $t_{0}=0$, $a(t)=|K_{0}|$, $b(t)=1$, $f_{1}(t)=\frac{3t}{1+3t}$, $f_{2}(t)=\frac {1}{t^{4}}$, $f_{3}(t)=e^{-t}$, $f_{4}(t)\equiv1$ $f_{5}(t)=f_{6}(t)\equiv0$, $g_{1}(t)=t^{2}$, $g_{2}(t)\equiv1$, $\alpha (t)=\frac{t}{2}$, $\beta(t)=\frac{t}{3}$, $p=\frac{1}{2}$, and $q=\frac {1}{3}$ in Theorem 3.1, on the basis of a straightforward computation, we obtain

$$\begin{aligned}& K = \int_{1}^{\frac{T}{3}}\frac{2}{3}k_{2}^{\frac{1}{3}}e^{-s} \,ds=\frac{2}{3}k_{2}^{\frac{1}{3}} \bigl(1-e^{-\frac{T}{3}} \bigr), \\& V(t) = |K_{0}|+ \int_{0}^{\frac{t}{2}}\frac{1}{2}k_{1}^{\frac{1}{2}} \frac{3s}{1+3s}\,ds =|K_{0}|+\frac{1}{2}k_{1}^{\frac{1}{2}} \biggl(\frac{t}{2}-\frac{1}{3}\ln \biggl(1+\frac{3t}{2} \biggr) \biggr), \\& A(t) = 0,\qquad B(t)=0,\qquad C(t)=\frac{1}{4}k_{1}^{-\frac{1}{2}}, \\& e_{B \oplus C}(t,0) = \exp \biggl\{ \frac{1}{4}k_{1}^{-\frac{1}{2}}s \biggr\} . \end{aligned}$$

Using Theorem 3.1, we obtain the desired inequality (4.7). □

## Conclusions

In this paper, we have established some new retarded Volterra–Fredholm type integral inequalities on time scales, which extend some known inequalities and provide a handy tool for deriving bounds of solutions of retarded dynamic equations on time scales. Unlike some existing results in the literature, the integral inequalities considered in this paper involve the power nonlinearity, which results in difficulties in the estimation on the explicit bounds of unknown function $u(t)$. We establish an inequality to overcome the difficulties, which can be used as a handy tool to solve the similar problems.
